# Empyema of the Antrum

**Published:** 1894-03-10

**Authors:** A. W. W. Hoffman


### EMPYEMA OF THE ANTRUM.

By A. W. W. Hoffman, M.R.C.S., L.D.S. Eng.

A great deal of attention has been given to empyema of the antrum, since Ziem published his first paper in 1886. There is now a special literature in connection with the disease; facts have been carefully collected, new operations and methods of treatment have been proposed and carried out. Still, at the present time, in spite of all the valuable work that has been done, our views concerning its origin are not as clear, and our efforts for its relief not as generally or as speedily successful, as we could wish.

The whole subject is still rather a vexed question. Authorities differ considerably as to its etiology, its diagnosis, and its treatment:

Suppuration in the antrum is, in almost every instance, secondary to inflammation arising in connection with the nose or with the teeth. On this point all are agreed. It is with regard to the relative frequency of infection, from one or other source that such a wide difference of opinion exists. On the one hand are those who consider that the source of inflammation is always, or nearly always, a dental one; on the other those who believe that it must be sought for in many cases in the nose. The subject is of considerable importance as having a very practical bearing on the treatment. As the antrum is lined by a mucous membrane continuous with that of the nose, and intimately connected with it by its blood supply, there is nothing more probable, than that inflammation should extend from one to the other; yet the bulk of evidence seems to point to the teeth, as the cause, in the great majority of cases.

Closely as the antrum is related to the nose, it is still more intimately related to the teeth. The roots of the first and second molars can almost always be seen causing rounded prominences on the floor of the cavity, occasionally even perforating it, covered only by periosteum and mucous membrane. In such cases inflammation of the tooth must necessarily extend to the antrum. It must be remembered that in these cases there may be no distinct history of dental trouble. Should an abscess occur in connection with such a tooth, only very transient pain may be felt before pressure is relieved by the pus escaping into the antrum; and the tooth may exhibit only slight signs of inflammation. Teeth are occasionally met with in which the pulp has died, and which are yet entirely free from caries, and such teeth are peculiarly liable to give rise to abscess. It must also be remembered that while disease of the nose is comparatively rare, disease of the teeth, and particularly of the first molar, is very common.

Bayer has noticed the frequency with which nasal polypi are associated with antral disease, but it does not seem clear if they are a cause or an effect of the condition. They may possibly be due to irritation of the nasal mucous membrane, by pus flowing over it.

Cases of empyema of the antrum naturally divide themselves in two classes—(1) Those in which the pus can flow from the antrum without obstruction into the nose, and in which there is a discharge of pus from the nostril. (2) Those in which the natural opening is blocked, either permanently or temporarily, and in which distension of the cavity occurs.

In the first class the most marked symptom will be the discharge of pus. Antral disease is not by any means the only cause of a purulent discharge from the nose, but the following characteristics will help to distinguish it from other morbid conditions: (1) It is unilateral, the pus coming from the nostril on the affected side. (2) It is intermittent. The flow of pus does not take place constantly, but only when the position of the patient is such as to favour its escape from opening of the antrum. Patients will complain that pus trickles from one nostril on stooping, or when lying down, particularly when lying on the sound side. During sleep the pus will run back into the throat and be swallowed, giving rise to a disagreeable taste in the mouth on waking, and, as was pointed out by Mr. Heath, causing a troublesome form of dyspepsia. (3) The pus is always fœtid, and the odour is perceived by the patient, though not always by other people. This is in marked contrast to the discharge of ozœna. Here the discharge is continuous, and the patient is almost unconscious of the disgusting odour, so perceptible to those around. In empyema, the smell, often forms the patient's chief cause of complaint.

*Pain* is not always a constant symptom. In a few cases none is felt. When present, it is generally dull and heavy in character, and is frequently referred to the frontal region. At times, if the free exit of pus is prevented, either by swelling of the soft parts or by the formation of crusts, severe neuralgic pains may be felt in the ear, the face, and the teeth of the upper jaw. It is very rarely referred to the seat of the disease. In very acute cases there may be some tenderness over the malar bone and slight œdema of the cheek. Even when there is no obstruction to the escape of pus there is usually some amount of blocking of the nostril. A general condition of ill-health, with considerable depression of spirits and troublesome dyspepsia, is not uncommon, especially if there is a copious discharge of pus.

When the escape of pus is prevented the antrum becomes gradually distended. It seems doubtful if the extreme distension met with at times could be produced by any obstruction of the opening arising from crusts or swelling. It is probable that many such cases (not due to cystic disease) are caused by a chronic alveolar abscess invading the antral cavity and dilating it, the dense sac wall effectually preventing any escape of pus into the nose. Under such circumstances very considerable deformity may be caused; the floor of the orbit pushed up, causing exophthalmos; the nostril completely blocked; the arch of the palate on the affected side lost, and a large swelling formed in the zygomatic fossa, and in the sulcus between the gum and the cheek. The teeth, though lengthened and tender, are very rarely displaced in antral disease.

Pain in such cases is usually very severe, though occasionally it may be entirely absent. Pressure over the swelling may give rise to egg-shell crackling of the thinned bone, but this is much more commonly found associated with cystic disease.

In many cases the diagnosis of antral disease is very simple; the presence of a characteristic discharge of pus from the nostril, associated with obviously diseased teeth, being almost conclusive. When the antrum is expanded we shall have to distinguish between empyema, a cyst of the antral wall, a dental cyst, and new growths, simple, and malignant. This is not usually a difficult matter, and in doubtful cases an exploratory puncture will decide the matter.

When we meet with a case in which the teeth are sound and in which the only symptom is a flow of pus from the nose, and when we remember that the pus may come from the antrum, or from one of the other accessory nasal cavities, or from both, and that ozœna and antral disease may exist together, it becomes a matter of the greatest difficulty to decide whether the antrum is diseased or not.

Percussion, catheterisation through the natural orifice, exploratory puncture from the mouth, or from the inferior meatus, have all been recommended in turn, and have proved more or less unsatisfactory; indeed the second expedient has been charged, not perhaps altogether without reason, with producing the very condition we are seeking to remedy.

Two other methods of diagnosis remain. If pus can be clearly seen coming from the ostium maxillare the question is of course decided; but this is by on means always an easy matter. The following mode of procedure is recommended in order to demonstrate this clearly. After thorough cocainisation the nostril is carefully cleansed, and the patient made to recline on the sound side for a few moments, the head being kept low. The speculum is now introduced, and we may hope to see a stream of pus running down the outer wall of the nose. If the discharge is very free it may be necessary to repeat the process, for with the nostril flooded with pus it is impossible to decide from whence it comes. At the second attempt the antrum will be so far emptied that only a small stream escapes, and its origin can be clearly made out.

Our powers of diagnosis have now been added to by the use of the electric light. Heryng recommends this method strongly. The patient is placed in a perfectly dark room, and a small incandescent lamp introduced into the mouth. It is found that through a healthy antrum a certain amount of light passes, but that the presence of pus renders it opaque.

(*To be continued.*)

